# Case Report: Hypomorphic Function and Somatic Reversion in DOCK8 Deficiency in One Patient With Two Novel Variants and Sclerosing Cholangitis

**DOI:** 10.3389/fimmu.2021.673487

**Published:** 2021-04-16

**Authors:** Francesco Saettini, Grazia Fazio, Daniele Moratto, Marta Galbiati, Nicola Zucchini, Davide Ippolito, Marco Emilio Dinelli, Luisa Imberti, Mario Mauri, Maria Luisa Melzi, Sonia Bonanomi, Alessio Gerussi, Marinella Pinelli, Chiara Barisani, Cristina Bugarin, Marco Chiarini, Mauro Giacomelli, Rocco Piazza, Giovanni Cazzaniga, Pietro Invernizzi, Silvia Clara Giliani, Raffaele Badolato, Andrea Biondi

**Affiliations:** ^1^ Pediatric Hematology Outpatient Clinic, Department of Pediatrics, Fondazione MBBM, Monza, Italy; ^2^ Centro Ricerca Tettamanti, University of Milano Bicocca, Monza, Italy; ^3^ Flow Cytometry Laboratory, Diagnostic Department, ASST Spedali Civili, Brescia, Italy; ^4^ Division of Pathology, San Gerardo Hospital, ASST Monza, Monza, Italy; ^5^ Department of Diagnostic Radiology, San Gerardo Hospital, Monza, Italy; ^6^ Endoscopy Unit, San Gerardo Hospital, ASST, Monza, Italy; ^7^ Centro di Ricerca Emato-oncologica AIL (CREA), ASST Spedali Civili, Brescia, Italy; ^8^ Department of Medicine and Surgery, University of Milano Bicocca and San Gerardo Hospital, Monza, Italy; ^9^ Department of Pediatrics, Fondazione MBBM, Monza, Italy; ^10^ Division of Gastroenterology, Centre for Autoimmune Liver Diseases, Department of Medicine and Surgery, University of Milano-Bicocca, Monza, Italy; ^11^ European Reference Network on Hepatological Diseases (ERN RARE-LIVER), San Gerardo Hospital, Monza, Italy; ^12^ Cytogenetic and Medical Genetic Unit, Department of Molecular and Translational medicine, A. Nocivelli Institute for Molecular Medicine, University of Brescia, Spedali Civili, Brescia, Italy; ^13^ Department of Clinical and Experimental Sciences, Pediatrics Clinic and A. Nocivelli Institute for Molecular Medicine A, University of Brescia, ASST-Spedali Civili, Brescia, Italy

**Keywords:** primary immumunodeficiencies, DOCK 8, EBV - Epstein-Barr Virus, sclerosing cholangitis, thrombocytopenia, lymphopenia, somatic reversion

## Abstract

DOCK8 deficiency is a combined immunodeficiency due to biallelic variants in dedicator of cytokinesis 8 (*DOCK8*) gene. The disease has a wide clinical spectrum encompassing recurrent infections (candidiasis, viral and bacterial infections), virally driven malignancies and immune dysregulatory features, including autoimmune (cytopenia and vasculitis) as well as allergic disorders (eczema, asthma, and food allergy). Hypomorphic function and somatic reversion of *DOCK8* has been reported to result in incomplete phenotype without IgE overproduction. Here we describe a case of DOCK8 deficiency in a 8-year-old Caucasian girl. The patient’s disease was initially classified as autoimmune thrombocytopenia, which then evolved toward a combined immunodeficiency phenotype with recurrent infections, persistent EBV infection and lymphoproliferation. Two novel variants (one deletion and one premature stop codon) were characterized, resulting in markedly reduced, but not absent, DOCK8 expression. Somatic reversion of the *DOCK8* deletion was identified in T cells. Hypomorphic function and somatic reversion were associated with restricted T cell repertoire, decreased STAT5 phosphorylation and impaired immune synapse functioning in T cells. Although the patient presented with incomplete phenotype (absence of markedly increase IgE and eosinophil count), sclerosing cholangitis was incidentally detected, thus indicating that hypomorphic function and somatic reversion of *DOCK8* may delay disease progression but do not necessarily prevent from severe complications.

## Introduction

The dedicator of cytokinesis 8 (DOCK8) protein exerts critical roles in both humoral and cellular immune responses. It mediates cell differentiation, survival, adhesion and migration, promoting the immune synapse (IS) by active cytoskeletal response (1–4).

DOCK8 deficiency was described for the first time in 2009 in patients with biallelic loss-of-function variants (1, 2). Patients with DOCK8 deficiency are prone to recurrent infections, including candidiasis, viral and bacterial infections, and virally driven malignancies. DOCK8 deficiency has been associated with a number of immune dysregulatory features, including autoimmune disorders (cytopenia and vasculitis), as well as allergic disorders (eczema, asthma, and food allergy). Blood tests reveal T-cell lymphopenia with hypereosinophilia. Serum immunoglobulin (Ig) levels usually show hyperIgE with low IgM. The current consensus is to perform allogenic hematopoietic stem cell transplantation (HSCT) as early as possible, due to high morbidity and premature mortality (5, 6). Patients with less severe phenotype have been subsequently reported (7, 8). In these individuals, hypomorphic function and somatic reversion of the mutated *DOCK8* allele have been identified and associated with a milder phenotype. Yet, the long-term prognosis of this subgroup of DOCK8-deficient patients does not seem to be ameliorated (7, 8) and the only curative treatment remains HSCT (5).

In this report, we describe a 8-year-old girl with two novel *DOCK8* variants (one nonsense variant and one deletion). Hypomorphic function and somatic reversion of *DOCK8* resulted in an incomplete phenotype and sclerosing cholangitis (SC) was incidentally diagnosed.

## Case Description

**Figure 1:**
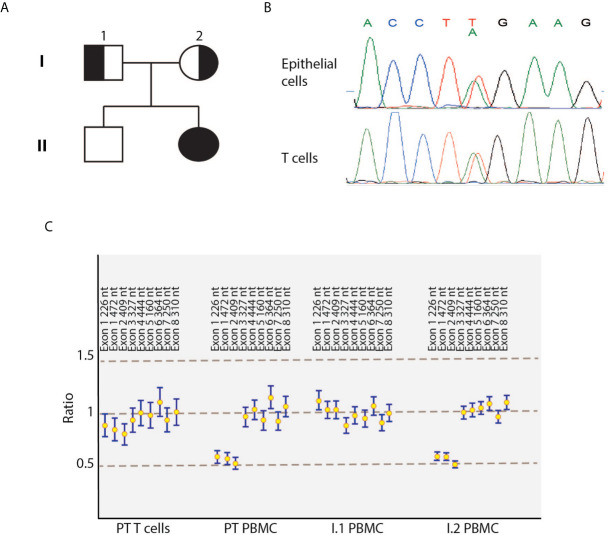
Identification of compound heterozygous *DOCK8* variants in the index patient. **(A)** Pedigree of the family. Solid symbols indicate affected patient; half solid symbols, heterozygous persons; circles, female family members; square, male family members. **(B)** Electropherograms showing the novel c.T824A mutation in *DOCK8* patient’s epithelial cells and T-cell blasts. **(C)** Reversion of the deletion in patient’s T cells blasts detected by multiplex ligation-dependent probe amplification. PBMC, peripheral blood mononuclear cells; PT, patient.

**Figure 2:**
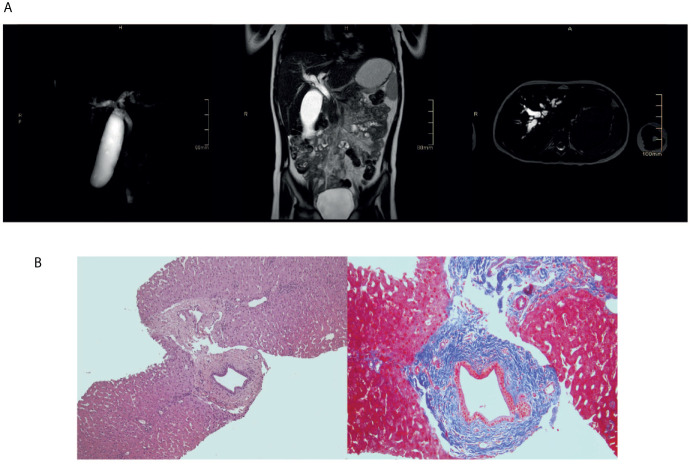
Sclerosing cholangitis in the index patient. **(A)** Upper abdomen magnetic resonance showing diffuse and marked enlargement of intra- and extrahepatic bile ducts. **(B)** Liver biopsy showing one dilated duct with “onion-skin” type of periductal fibrosis.

The index patient was a 8-year-old female patient born from non-consanguineous parents (**Figure 1A**). Eczema started at the age of 6 months and it was treated with topical steroids and emollients. Recurrent skin bacterial infections occurred, but they responded well to topical antibiotics. At the age of two, she had two episodes of parotitis treated with intravenous antibiotics. The second one was complicated by cellulitis. Vulvovaginal candidiasis occurred once, along with abscess of the outer labia. At the age of three, she had thrombocytopenia in the setting of active EBV infection (9). Platelet count did not increase with high doses of intravenous immunoglobulin (IVIg), requiring oral steroids. Thereafter, EBV DNA remained detectable with viral load ranging from 2 x 10^2^ to 5 x 10^3^ copies/ml. During the follow-up, persistent lymphadenopathies and lymphopenia were detected. Hypereosinophilia was never observed. IgE, initially within normal range, mildly increased by the age of 6 (**Table 1**). Upper and lower respiratory tract infections were frequent. The most common bacterium in sputum was *Haemophilus Influenzae*. Bacteria were always sensitive to commonly-used antibiotics. Antimicrobial, antiviral, and antifungal prophylaxis and monthly IVIg infusion were started with good clinical improvement. Lung computed tomography performed at the age of six showed diffuse bronchial wall thickening with tree-in-bud opacities and mediastinal adenopathies. Incidentally, marked enlargement of the biliary tree was noticed, which was absent at a previous abdominal ultrasound. Liver test were normal (including alanine transaminase, aspartate transaminasase, gamma-glutamyltransferase, prothrombin time, and total bilirubin) except for mildly increased bile acids. Testing for infectious agents, including *Rotavirus*, *Adenovirus*, *Clostridium Difficile*, *Giardia*, and *Cryptosporidium* was negative. With respect to Cryptosporidium, fecal samples were repeatedly tested by means of microscopic analysis and chromatographic immunoassay for the detection of *Cryptosporidium* antigens. Marked dilation of intrahepatic bile ducts (more pronounced at the right system) and dilation of the common bile duct (up to 7 mm and without any mass forming lesion in the lumen) were then confirmed by means of abdominal ultrasound and magnetic resonance imaging (**Figure 2A**). Liver biopsy showed one dilated duct with “onion-skin” type of periductal fibrosis and portal and acinar inflammation consistent with SC (**Figure 2B**). Fecal calprotectin was mildly increased (185 μg/mg; normal up to 50 μg/mg). Upper gastroenterology endoscopy showed enlarged duodenal papilla. Histology revealed moderate chronic gastritis and mild chronic inflammation of the duodenal mucosa. Colonoscopy showed mild mucosal inflammation with mixed cell infiltrate, including macrophages, eosinophils, and plasma cells. Considering the good clinical condition, endoscopic retrograde cholangiopancreatography with endoscopic sphincterotomy of the major duodenal papilla was successfully performed with disposable duodenoscope (10) and no immunosuppressive treatment was initiated. Patient remained well, without any abdominal symptom.

**Table 1:** Immunological characteristics of the patient.

	3 years	4 years	5 years	6 years	7 years
Hemoglobin, g/dl	11.1 (11.5-13.5)	12.1 (11.5-13.5)	12.5 (11.5-13.5)	11.9 (11.5-15.5)	12.3 (11.5-15.5)
WBC, x 10^9^/l	3.93 (5.2-11.0)	6.26 (5.2-11.0)	7.35 (5.2-11.0)	7.35 (4.4-9.5)	2.62 (4.4-9.5)
PLT, x 10^9^/l	25 (>140)	330 (>140)	337 (>140)	423 (>140)	332 (>140)
Neutrophils, x 10^9^/l	1.05 (>1.5)	4.34 (>1.5)	4.94 (>1.5)	5.0 (>1.5)	1.1 (>1.5)
Lymphocytes, x 10^9^/l	2.28 (2.3-5.4)	1.08 (2.3-5.4)	1.52 (2.3-5.4)	1.23 (1.9-3.7)	0.61 (1.9-3.7)
Eosinophils, x 10^9^/l	0.14 (<0.5)	0.27 (<0.5)	0.17 (<0.5)	0.22 (<0.5)	0.33 (<0.5)
IgG, mg/dl	1971* (462-1710)	643 (528-1959)		823 (633-1919)	913 (633-1919)
IgA, mg/dl	130 (27-173)	93 (37-257)		75 (41-315)	40 (41-315)
IgM, mg/dl	43 (62-257)	18 (49-292)		39 (56-261)	13 (56-261)
IgE, kU/l	19	25		493	504
CD3^+^, x 10^9^/l	1.05 (1.4-3.7)	0.62 (1.4-3.7)	0.85 (1.4-3.7)	0.73 (0.99-3.53)	0.60 (0.99-3.53)
CD4^+^, x 10^9^/l	0.43 (0.7-2.2)	0.39 (0.7-2.2)	0.44 (0.7-2.2)	0.36 (0.50-2.12)	0.41 (0.50-2.12)
Naïve CD4^+^CD45RA^+^CCR7^+^, %		42.2 (49.2-85.8)	28.6 (37.8-80.3)	40.4 (37.8-80.3)	35.0 (37.8-80.3)
Recent thymic emigrants CD4^+^CD45RA^+^CCR7^+^CD31^+^, x 10^9^/l		32.0% (36.2-71.8)	21.8% (20.3-68.9)	115 (190-1024)	118 (190-1024)
Central Memory CD4^+^CD45RA^-^CCR7^+^, %		33.8 (9.6-31.9)	38.1 (9.9-41.1)	27.4 (9.9-41.1)	12.0 (9.9-41.1)
Effector Memory CD4^+^CD45RA^-^CCR7^-^, %		21.8 (2.8-16.9)	30.9 (4.0-25.5)	30.6 (4.0-25.5)	31.8 (4.0-25.5)
Terminally Differentiated CD4^+^CD45RA^+^CCR7^-^, %		2.3 (0.7-4.8)	2.8 (0.4-7.7)	1.5 (0.4-7.7)	21.2 (0.4-7.7)
Regulatory T cells CD4^+^CD25^+^CD127^low/-^, x 10^3^/l					21 (42-207)
Regulatory T cells CD4^+^CD25^+^CD127^low/-^, %					5.0 (7.0-17.3)
CD8^+^, x 10^9^/l	0.44 (0.49-1.3)	0.15 (0.49-1.3)	0.31 (0.49-1.3)	0.28 (0.25-1.34)	0.13 (0.25-1.34)
Naïve CD8^+^CD45RA^+^CCR7^+^, %		8.9 (22.8-79.9)	4.1 (20.3-78.2)	5.3 (20.3-78.2)	12.7 (20.3-78.2)
Central Memory CD8^+^CD45RA^-^CCR7^+^, %		4.7% (0.9-11.3)	3.2% (1.7-13.3)	0.9% (1.7-13.3)	2.2% (1.7-13.3)
Effector Memory CD8^+^CD45RA^-^CCR7^-^, %		37.1% (4.3-31.4)	40.8% (8.6-34.5)	41.9% (8.6-34.5)	25.5% (8.6-34.5)
Terminally Differentiated CD8^+^CD45RA+CCR7-, %		49.7% (6.8-52.7)	52.3% (7.0-53.8)	51.8% (7.0-53.8)	59.6% (7.0-53.8)
CD19^+^, x 10^9^/l	0.97 (0.39-1.4)	0.39 (0.39-1.4)	0.53 (0.39-1.4)	0.43 (0.22-1.13)	0^+^
Recent B emigrants CD19^+^CD38^++^CD10^+^, %		19.5% (10.6-42.6)	16.9% (7.1-35.3)	28.8% (7.1-35.3)	
Naïve CD19^+^IgD^+^IgM^+^CD27^-^, %		66.0% (34.2-65.5)	61.0% (37.1-70.2)	55.5% (37.1-70.2)	
CD19^++^CD21^low^, %		8.1% (1.5-9.8)	9.8% (1.9-9.0)	10.7% (1.9-9.0)	
Switched Memory CD19^+^IgD^-^IgM^-^CD27^+^, %		0.3% (1.5-4.2)	0.6% (2.4-19.8)	0.7% (2.4-19.8)	
IgM Memory CD19^+^IgD^+^IgM^+^CD27^+^, %		1.9% (2.9-15.3)	3.5% (3.1-18.3)	1.6% (3.1-18.3)	
Terminally differentiated CD19^+^CD38^++^CD27^+^CD20^-^, %		2.2% (0.4-15.3)	4.9% (0.3-11.8)	1.7% (0.3-11.8)	
CD3^-^CD16^+^CD56^+^, x 10^9^/l	0.09 (0.13-0.72)	0.05 (0.13-0.72)	0.10 (0.13-0.72)	0.1 (0.08-1.03)	0.02 (0.08-1.03)

* After IVIg. ^+^After rituximab therapy.

Age-matched normal values are given between round brackets.

During the follow-up, neutropenia was detected with occasionally normal neutrophil count (**Table 1**). Analyses of main lymphocyte population consistently displayed reduced T cell counts compared to age-matched healthy controls (HC), while evaluation of lymphocyte subsets revealed a moderate reduction of naïve T cells and memory B cells. At 7 years of age, regulatory T cells (T_reg_), defined as CD4+CD25+CD127^low/-^ cells, were analyzed, resulting in a percentage that was below the detection limit of age-matched HC and in a significant reduction of their absolute count (below 2SD from mean; **Table 1**). The release of new lymphocytes by the thymus, either quantified by means of T-cell receptor (TR) excision circles (TRECs) or recent thymic emigrants’ counts, was reduced in comparison to that of age-matched HC. Flow cytometric analysis of T lymphocyte proliferation by incorporation of carboxyfluorescein diacetate succinimidyl ester was assessed, displaying a markedly reduced proliferation ability at the level of CD8^+^ cells either upon stimulation with phytoemoagglutinin (PHA) or with anti-CD3 monoclonal antibody. Spectratyping analysis of T-cell receptor variable beta (TRBV) chain subgroups revealed a restricted T-cell repertoire. The production of new B-lymphocytes from the bone marrow, measured by means of K-deleting recombination excision circles (KRECs), was comparable to that HC. Evaluation of serum immunoglobulins indicated reduced IgM and moderately elevated IgE (**Table 1**).

**Figure 3:**
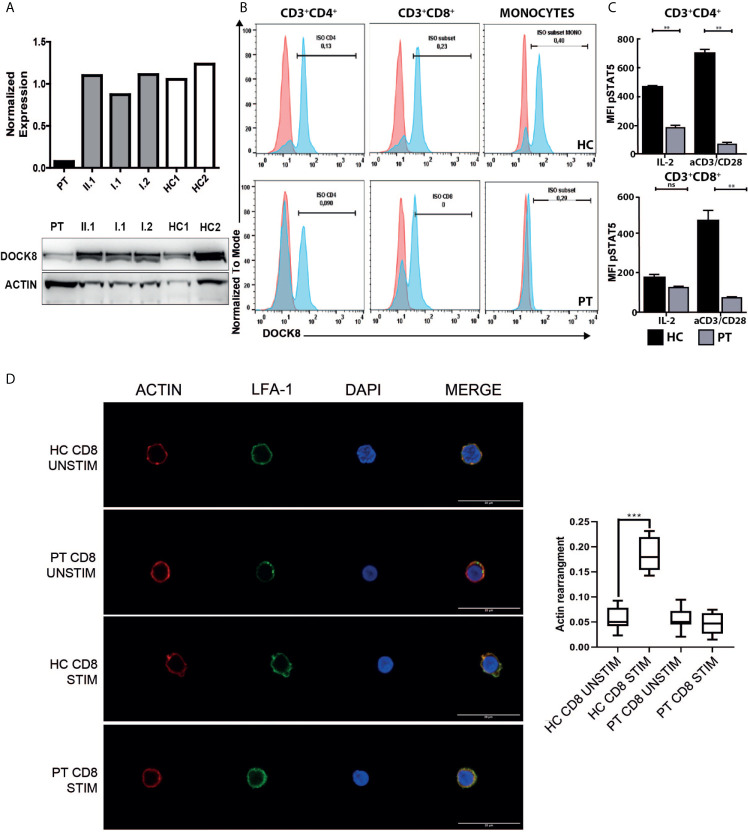
Functional characterization of the *DOCK8* variants. **(A)** Evaluation of DOCK8 levels in IL2-PHA expanded T-cell of the index patient, family members and two healthy controls (HC1 and HC2) by means of flow cytometry (upper panel; one representative experiment of 2 independent experiments) and western blot (lower panel; one representative experiment of 2 independent experiments). **(B)** Flow cytometric analysis of DOCK8 expression in peripheral T-cells and monocytes of the patient and a healthy control, showing hypomorphic DOCK8 expression in CD3^+^CD4^+^ and CD3^+^CD8^+^. (representative experiment) **(C)** Reduction of STAT5 phosphorylation in patient’s peripheral CD4^+^ and CD8^+^ T-cells at baseline and following stimulation with IL2 or CD3/CD28 monoclonal antibodies. (one representative experiment performed in duplicates) **(D)** Representative images of Actin (Red), LFA-1 (green) and DAPI (Blue) staining on unstimulated or CD3/CD28 stimulated CD8^+^ cells from the index patient and a healthy donor (representative experiment). Scale bar: 20um. In the right panel, box plots show the quantification of actin signal in analyzed samples. (**p < 0.01; ***p < 0.001). HC, healthy control; PT, patient; Stim, stimulated; Unstim, unstimulated. NS, not significant.

Considering the clinical manifestations and the immunology evaluation, combined immunodeficiency was suspected, and molecular analysis performed. At the age of seven, using targeted next generation sequencing (NGS) and multiplex ligation-dependent probe amplification (MLPA), we identified distinct biallelic variants in *DOCK8*, which were not present in public databases (gnomAD, ESP, and 1000 Genomes) and were exclusive to this family in our internal databases. NGS revealed a heterozygous NM_203447:c.T824A variant in exon 7 of the *DOCK8* gene, resulting in a premature stop codon (p.L275*), which was confirmed by Sanger sequencing (**Figure 1B**). The father was heterozygous carrier of the variant. Single nucleotide polymorphism (SNP) array and MLPA revealed the maternally inherited deletion of exons 1 to 3 (**Figure 1C**). Both variants are supposed for their nature to lead to a null DOCK8 phenotype being pathogenetic (class 5). Flow cytometric and western blot analysis showed markedly reduced, but not absent, DOCK8 expression in patient’s interleukin-2 (IL2)-PHA expanded T-cells, CD3^+^CD4^+^ and CD3^+^CD8^+^ while DOCK8 expression was absent in monocytes. These results collectively suggested hypomorphic residual function in T-cells (**Figures 3A, B**). Sanger sequencing analysis performed on epithelial cells as well as on peripheral T-cells confirmed the presence of the heterozygous status for the T824A variant in all the lineages, excluding the possibility of somatic reversion for this variant, while a somatic reversion of the deletion was detected by MLPA analysis in patient’s T-cells blasts (**Figures 1B, C**). STAT5 phosphorylation in T-cells (both CD3^+^CD4^+^ and CD3^+^CD8^+^) was reduced either after anti-CD3/CD28 or IL-2 stimulation (**Figure 3C**). To examine the role of the novel variants in the IS, CD8^+^ were stimulated with anti-CD3 and anti-CD28 monoclonal antibodies to mimic the interaction between CD8^+^ cells and dendritic cells. Patient’s cells exhibited a significantly reduced actin polymerization in response to stimuli, whose distribution pattern was similar to the one displayed by LFA-1 (**Figure 3D**).

Haploidentical related donor HSCT was performed at 7 years and 6 months of age. Due to donor specific anti-human leukocyte antigen antibodies, desensitization protocol included a single dose of 375 mg/m^2^ rituximab and plasma exchange. Conditioning regimen included 2 days of low dose cyclofosmamide, 5 days of fludarabin, 3 days of busulfan and 200 cGy total body irradiation. Graf-versus-host disease (GVHD) prophylaxis consisted of cyclosporine (from day -1, ongoing), cyclofosfamide (on day +3 and +4) and mycophenolate (from day +1 to day +26). Neutrophil engraftment (0.5 x 10^9^/l) occurred at day +17 (1.0 x 10^9^/l at day +19) and platelet engraftment (20 x 10^9^/l) occurred on day +18 (50 x 10^9^/l at day +19). Complete donor chimerism was detected on day +25 and thereafter checked every 14 days. Main complication after HSCT was hyperacute stage 2 grade 1 skin GVHD on day +16, which required additional therapy with methylprednisolone at 1 mg/kg/day and resolved on day +18. CMV reactivation on day +34 was successfully treated with valganciclovir. The patient is now alive and well 122 days after HSCT, with no signs of GVHD. Chimerism will be checked every 14 days until day +180 and afterward once a month until 1 year after HSCT.

## Discussion

The variant spectrum of inborn errors of immunity (IEI)/primary immunodeficiencies (PID) comprises unconventional genetic etiologies. Large deletions have been frequently reported in DOCK8-deficient patients (11), thus requiring including copy number variants (CNV) analysis in patients with complex phenotypes (12). If whole exome/whole genome sequencing with CNV analysis cannot be performed, a combination of NGS, SNP array, or MLPA should be used.

The low-level DOCK8 protein expression in patient’s T cells suggests either residual protein synthesis by means of alternative splicing (13) or somatic reversion (7, 8). Somatic reversions have been observed in several immunodeficiencies (i.e. Wiskott Aldrich syndrome) (14). In DOCK8 deficiency this phenomenon is enriched in T cells, particularly antigen-experienced T cells. Chronic antigenic stimulation, especially viral, contributes to the expansion of such subgroup of T cells. It has been proposed that reversion may confer a selective advantage and may correct Th2 skewing due to abnormal cytokine production thereby modulating disease’s phenotype (7). This phenomenon, nonetheless, does not ameliorate the defective formation of the IS in CD8^+^ T lymphocytes (4). Although hypormorphic DOCK8-deficient patients may present with an incomplete phenotype (i.e. lack of markedly elevated IgE levels, hypereosinophilia, and/or severe viral skin infections), patients still experienced disease progression and severe or fatal complications (7, 8). Interestingly, while preparing this manuscript, clinical improvement over time has been described in 3 DOCK8-deficient patients (15). Authors demonstrated that somatic reversion of one of the mutated alleles restored lymphocyte survival, differentiation and function. We did not assess revertant T and B subsets, but we show that the qualitative defect in CD4^+^ and CD8^+^ was not reverted. In our patient, *in vitro* stimulation with anti-CD3 or PHA, T-cell repertoire, STAT5 phosphorylation and IS polarization were impaired (4, 16). In keeping with this, the frequency of infectious episodes decreased only when prophylaxes and IVIg had been started. Therefore, despite our patient showed spontaneous repair, we decided to perform HSCT.

As noted in the larger DOCK8 cohorts so-far described (3, 4), some of our patient’s manifestations could overlap with other IEI presenting with hypogammaglobulinemia and lymphoproliferation (17), thrombocytopenia (9), lymphopenia with low but detectably persistent EBV copies (18), and occasionally normal neutrophils count (19). Some forms of PID/IEI have a high-incidence of liver disease and SC, i.e. common variable immunodeficiency and hyperIgM syndrome. Up to 70% of them are colonized with *Cryptosporidium parvum*, and this has been associated with significant chronic liver disease (20, 21). In case of abnormal liver function tests results, *Cryptosporidium* should be always ruled out in DOCK8-deficient patients, as it is a under-recognized cause of liver disease. Ideally, *Cryptosporidium* should be investigated by means of PCR, as it has been proven to be more sensitive than microscopic analysis and chromatographic immunoassay (22). In a large cohort of DOCK8-deficient patients, SC has been reported in 5% of patients but authors described no autoimmune gastrointestinal manifestations (6). Subsequently, IPEX-like phenotype and active colitis have been associated with DOCK8 deficiency (13, 23). Autoimmune phenomena, in our case SC, have been related to defective number and function of T_reg_, depending on DOCK8 deficiency (15, 23).

With this report, we suggest that SC can be incidentally detected even in asymptomatic patients with apparently mild phenotype due to hypormorphic DOCK8 function. Regular biochemical and radiological follow up should be scheduled even in patients with no history of liver disease. These findings can lead to personalized follow-up and to early intervention in case of evolution of the manifestations. Larger studies focusing on hypomorphic DOCK8-deficient patients should investigate the immunological differences in individuals with clinical improvement over time compared to patients who still experience severe complications.

## Data Availability Statement

The raw data supporting the conclusions of this article will be made available by the authors, without undue reservation.

## Ethics Statement

Written informed consent was obtained from the legal guardian for the publication of any potentially identifiable images or data included in this article.

## Author Contributions

FS and RB contributed to conception and design of the study. FS wrote the first draft of the manuscript. All authors contributed to the article and approved the submitted version.

## Conflict of Interest

The authors declare that the research was conducted in the absence of any commercial or financial relationships that could be constructed as a potential conflict of interest.
