# Supplementary material for: Case Report: Hypomorphic Function and Somatic Reversion in DOCK8 Deficiency in One Patient With Two Novel Variants and Sclerosing Cholangitis
Source: Front Immunol. 2021 Apr 16;12:673487. doi: 10.3389/fimmu.2021.673487 (PMC8085392; doi:10.3389/fimmu.2021.673487)
Supplement: Supplementary file 1 [file DataSheet_1.docx]

**Hypomorphic function and somatic reversion in DOCK8 deficiency in one patient with two novel variants and sclerosing cholangitis**

Saettini F^1^, Fazio G^2^, Moratto D^3^, Galbiati M^2^, Zucchini N^4^, Ippolito D^5^, Dinelli ME^6^, Imberti L^7^, Mauri M^8^, Melzi ML^9^, Bonanomi S^1^, Gerussi A^10,11^, Pinelli M^12^, Barisani C^12^, Bugarin C^2^, Chiarini M^3^, Giacomelli M^12^, Piazza R^8^, Cazzaniga G^2,8^, Invernizzi P^10,11^, Giliani S^12^, Badolato R^13^, Biondi A^1,2^.

**Affiliations**

1. Pediatric Hematology Outpatient Clinic, Department of Pediatrics, Fondazione MBBM, Monza, Italy.

2. Centro Ricerca Tettamanti, University of Milano Bicocca, Monza, Italy.

3. Flow cytometry Laboratory, Diagnostic Department, ASST Spedali Civili, Brescia, Italy.

4. Division of Pathology, San Gerardo Hospital, ASST Monza, Monza, Italy.

5. Department of Diagnostic Radiology, San Gerardo Hospital, Monza, Italy.

6. Endoscopy Unit, San Gerardo Hospital, ASST Monza, Italy.

7. Centro di Ricerca Emato-oncologica AIL (CREA), ASST Spedali Civili, Brescia, Italy.

8. Department of Medicine and Surgery, University of Milano Bicocca and San Gerardo hospital, 20900, Monza, Italy.

9. Department of Pediatrics, Fondazione MBBM, Monza, Italy.

10. Division of Gastroenterology, Centre for Autoimmune Liver Diseases, Department of Medicine and Surgery, University of Milano-Bicocca, Monza, Italy.

11. European Reference Network on Hepatological Diseases (ERN RARE-LIVER), San Gerardo Hospital, Monza, Italy.

12. A. Nocivelli Institute for Molecular Medicine, Department of Molecular and Translational medicine, University of Brescia, Italy and Cytogenetic and Medical Genetic Unit, Spedali Civili, Brescia, Italy.

13. Pediatrics Clinic and A. Nocivelli Institute for Molecular Medicine A, Department of Clinical and Experimental Sciences, University of Brescia, ASST-Spedali Civili, Brescia, Italy.

**Corresponding author:** Francesco Saettini, Pediatric Hematology Department, Fondazione MBBM, Monza, Italy. Mail: [f.saettini@gmail.com](mailto:f.saettini@gmail.com) Phone: +390392333529

**Methods**

**Flow cytometric analyses**

Flow cytometric analyses were performed on fresh peripheral blood samples which were stained according to manufacturer’s protocols by using appropriate mixtures of monoclonal antibodies (MoAbs, all from BD Bioscience) directed against surface markers: CD3 (UCHT1), CD4 (RPA-T4), CD8 (SK1), CD16 (3G8), CD56 (NCAM16.2), CD19 (SJ25C1), HLA-DR (G46-6), CD45 (2D1) for analysis of main lymphocyte populations, while CD45RA (HI100), CCR7 (150503), CD31 (WM59), CD25 (2A3), CD127 (hIL-7R-M21), CD3 (UCHT1), CD4 (RPA-T4), CD8 (SK1), and CD10 (HI10a), CD19 (SJ25C1), CD20 (L27), CD21 (B-ly4), CD27 (L128), sIgD (IA6-2), sIgM (G20-127), CD38 (HIT2), for analysis of T- and B-cell subsets, respectively. Stained samples were acquired on a Canto II (BD Bioscience) flow cytometer and analyzed using DIVA software version 8.0.2.

**Analysis of proliferation and *In vitro* expansion of T-cells**

Evaluation of T-cell proliferation was performed after isolation of peripheral blood mononuclear cell (PBMC) using a Ficoll density gradient. PBMC were stained with carboxyfluoresecein diacetate succinimidyl ester (Becton Dickinson) according to manufacturer protocol and stimulated with coated anti-CD3 (5 μg/mL) (Beckman Coulter), anti-CD3+IL2 (600U/mL) (Sigma-Aldrich) or PHA (5 ng/μL) (Sigma-Aldrich). After four days of incubation cells were stained with anti-CD3 (UCHT1), anti-CD4 (RPA-T4), and anti-CD8 (SK1) MoAb (all from BD Bioscience); samples were acquired on a Canto II (BD Bioscience) flow cytometer and analyzed for T-cell proliferation using FlowJo software (TreeStar) version v10.

**DOCK8 expression**

***Cell culture***

PBMCs samples were isolated using the standard method of Ficoll Hypaque density gradient centrifugation. T cells were expanded by culturing at 5x10^5^ cells/mL in 96 well round bottom plates with RPMI 1640 media supplemented with 10% FCS and enhanced with PHA 5 μg/ml (Sigma Aldrich) and IL-2 600 U/ml (PeproTech). Cells were maintained in complete medium enhanced with IL-2 1200 U/ml.

***Cytometric analyses***

IL2-PHA expanded T-cells were collected, washed twice in PBS and suspended at the concentration of 1x10^6^ cell/ml per tube. Cells were stained with anti-CD3 PerCP-cy5.5 (BD Bioscience).

Mononuclear cells from peripheral blood were purified over Ficoll gradient. Cells were stained with surface antibodies to identify the following subsets: CD4^+^ T cells (CD4^+^), CD8^+^ T cells (CD8^+^) and monocytes (CD14^+^). All antibodies were obtained from BD Biosciences or from Biolegend. For multicolor flow analyses, CD3 conjugated to phycoerythrin, CD4 conjugated with APC, CD8 conjugated with APC and CD14 conjugated to PE were used (BD Bioscience).

After fixation and permeabilization according to the manufacturer's instructions (Nordic MUbio, Permeabilization solution), indirect staining was performed with either mouse anti-DOCK8 (Abcam ltd) or Rabbit IgG isotype control (Abcam Ltd), followed by secondary detection with Goat anti-Rabbit IgG-Alexa Fluor 488 (Abcam Ltd).

Flow cytometry was performed on an BD FACSCalibur, and data were analyzed with Flowjo software (Treestar, Ashland, Ore).

***Western blots***

Analysis of DOCK8 expression was performed on 2-week IL2-PHA expanded T-cells (7x10^6^ cells).

Separation and transfer of proteins, and immunoblotting for DOCK8 proteins were performed as described^1^. Blots were probed using rabbit anti-DOCK8 (Abcam ltd) and mouse anti-actin antibodies (Sigma-Aldrich).

1. Zhang Q, Davis JC, Lamborn IT, Freeman AF, Jing H, Favreau AJ, et al. Combined immunodeficiency associated with DOCK8 mutations. N Engl J Med 2009; 361:2046-55.

**Phosphoflow cytometry**

Mononuclear cells from patient and healthy donor were starved in X-VIVO medium and rested at 37°C for 1 hour thereafter cells were assessed for count and viability with trypan blue dye before phosphoflow testing. Cells from DOCK8 patient and healthy controls were stimulated with IL2 (10 ng/ml) for 15 minutes or with cross-linked anti CD3/CD28 mAbs following instruction of BD Phosflow™ Protocols for TCR Stimulation. Lymphocytes were identified using CD3 PERCP (Biolegend), CD4 PE-Cy7 (BD), CD8 FITC. Phosphorylation of Stat5 Y694 Alexa 488 (BD) was measured as MFI in CD3^+^CD4^+^ and CD3^+^CD8^+^ subpopulations.

Cells were acquired on a FACSaria™ flow cytometer (BD) equipped with 488-nm, 633-nm and 405-nm lasers. Data (at least 100,000 events per tube) were collected and analyzed using the DIVA™ software (BD). Statistical analysis and graph were analyzed using GraphPad Prism6 (GraphPad Software, Inc.). Statistics was performed using a one-sample t-test and data were expressed as means ± SD.

**CNV analysis**

Patient and parents' DNA samples were genotyped by CytoScan HD Array (Thermo Fisher Scientific, [Waltham, Massachusetts](https://www.google.com/search?client=firefox-b-d&sxsrf=ALeKk00FiWfZlftwOojSU9ldBnJGcGjZ2w:1615471547991&q=Waltham&stick=H4sIAAAAAAAAAOPgE-LSz9U3MCooMTBJU-IAsTOqjE21tLKTrfTzi9IT8zKrEksy8_NQOFYZqYkphaWJRSWpRcWLWNnDE3NKMhJzd7AyAgDThZNCUQAAAA&sa=X&ved=2ahUKEwjSz4y-tKjvAhVJy6QKHT7tDO4QmxMoATAaegQIHBAD), USA), according to the manufacturer’s protocol.

Analysis of copy number variations on DOCK8 region was performed with the Chromosome Analysis Suite software v. 4.2.0.80 (Thermo Fisher Scientific, [Waltham, Massachusetts](https://www.google.com/search?client=firefox-b-d&sxsrf=ALeKk00FiWfZlftwOojSU9ldBnJGcGjZ2w:1615471547991&q=Waltham&stick=H4sIAAAAAAAAAOPgE-LSz9U3MCooMTBJU-IAsTOqjE21tLKTrfTzi9IT8zKrEksy8_NQOFYZqYkphaWJRSWpRcWLWNnDE3NKMhJzd7AyAgDThZNCUQAAAA&sa=X&ved=2ahUKEwjSz4y-tKjvAhVJy6QKHT7tDO4QmxMoATAaegQIHBAD), USA) and based on hg19 assembly.

**Actin rearrangment**

CD8^+^ cells were FACS sorted from pheripheral blood obtained from the patient and an healty donor control.

After sorting cells were stimulated with anti CD3/CD28 mAbs for 30 minutes in culture condition, and subsequently fixed for 15 minutes at room temperature with 4% paraformaldehyde in 0.12M sodium phosphate buffer.

Cells were subsequently stained for 1 hour at RT with Phalloidin conjugated with Alexa Fluor 594 (Thermo fisher cat A12381), anti LFA-1 (CD18) FITC conjugated (Santa Cruz #SC-7306) and DAPI for nuclear counterstaining. Cells were washed twice in PBS and mounted on glass slides with a 90% (v/v) glycerol/PBS solution.

***Images acquisition and analysis***

Images were acquired using Zeiss LSM 710 confocal laser-scanning microscope (Zeiss) using a 63x, 1.4 N/A oil-immersion objective. Laser intensities and acquisition parameters were held constant throughout each experiment. Confocal microscopy fields were analyzed using specific homemade-designed macro with ImageJ (https://imagej.nih.gov/ij/) software. Briefly, for actin rearrangement measurements cells were sectioned in 4 constant defined quadrants and for each was measured the integrated density and calculated the standard deviation as index of homogeneity distribution. All data obtained derived from ten cells per samples.

Statistical analysis and graph were analyzed using GraphPad Prism8 (GraphPad Software, Inc.). Statistics was performed with ANOVA test and data were expressed as means ± SD.
